# Highly Ordered N-Heterocyclic Carbene Monolayers on Cu(111)

**DOI:** 10.1021/acs.jpclett.1c04073

**Published:** 2022-02-24

**Authors:** Eloise Angove, Federico Grillo, Herbert A. Früchtl, Alex J. Veinot, Ishwar Singh, J. Hugh Horton, Cathleen M. Crudden, Christopher J. Baddeley

**Affiliations:** † EaStCHEM School of Chemistry, University of St. Andrews, North Haugh, St Andrews, Fife KY16 9ST, United Kingdom; ‡ Department of Chemistry, Queen’s University, 90 Bader Lane, Kingston, Ontario Canada, K7L 3N6; § Institute of Transformative Bio-Molecules, ITbM-WPI, Nagoya University, Nagoya, Chikusa 464-8601, Japan

## Abstract

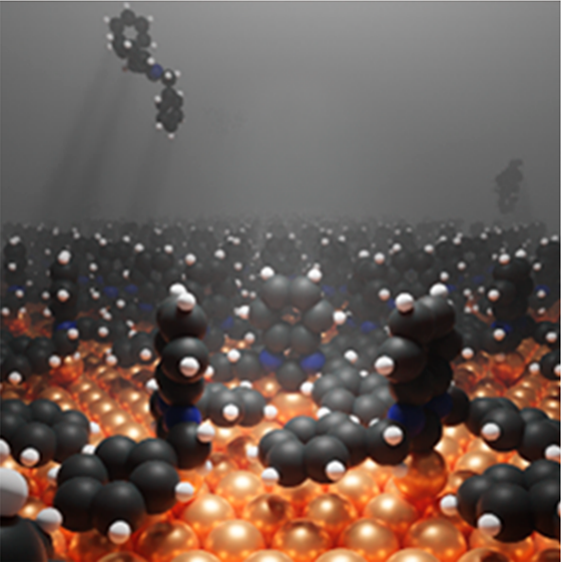

The benzannulated N-heterocyclic carbene, 1,3-dibenzylbenzimidazolylidene (NHC^DBZ^) forms large, highly ordered domains when adsorbed on Cu(111) under ultrahigh vacuum conditions. A combination of scanning tunnelling microscopy (STM), high-resolution electron energy loss spectroscopy (HREELS), and density functional theory (DFT) calculations reveals that the overlayer consists of vertical benzannulated NHC moieties coordinating to Cu adatoms. Long-range order results from the placement of the two benzyl substituents on opposite sides of the benzimidazole moiety, with their aromatic rings approximately parallel to the surface. The organization of three surface-bound benzyl substituents from three different NHCs into a triangular array controls the formation of a highly ordered Kagome-like surface lattice. By comparison with earlier studies of NHCs on Cu(111), we show that the binding geometry and self-assembly of NHC^DBZ^ are influenced by intermolecular and adsorbate–substrate interactions and facilitated by the flexibility of the methylene linkage between the N-heterocycle and the aromatic wingtip substituents.

N-heterocyclic carbenes (NHCs) are an emerging class of ligands for functionalizing extended metal surfaces, nanoparticles and nanoclusters.^1−18^ The ability of NHCs to produce self-assembled monolayers on a range of metallic, non-metallic, and metalloid substrates has attracted considerable interest with potential applications in multiple fields including catalysis, microelectronics, biosensing, surface protection, and 3D MOF architectures.^1,2,4,10,14,19−23^ In such applications, it is critical to control the orientation and packing of NHC monolayers to fine-tune surface density, metal accessibility, and ligand orientation. Although the factors dictating binding orientation and self-assembly are not fully elucidated, several studies have pointed toward the important effect of exocyclic nitrogen substituents (wingtips).^6,13,15,24−28^ Sterically congested N-substituents, such as iPr, tBu, Mes, and Dipp (wingtip groups), allow access to geometries whereby the heterocycle is perpendicular to the surface. As summarized in Scheme 1, top, regardless of their backbone structures, NHCs with primary substituents including Me, Et, and Bu give ordered overlayers composed of flat-lying M(NHC)_2_ species resulting from the abstraction of a metal atom (M) from the surface.^6,13,25−28^

**Scheme 1:**
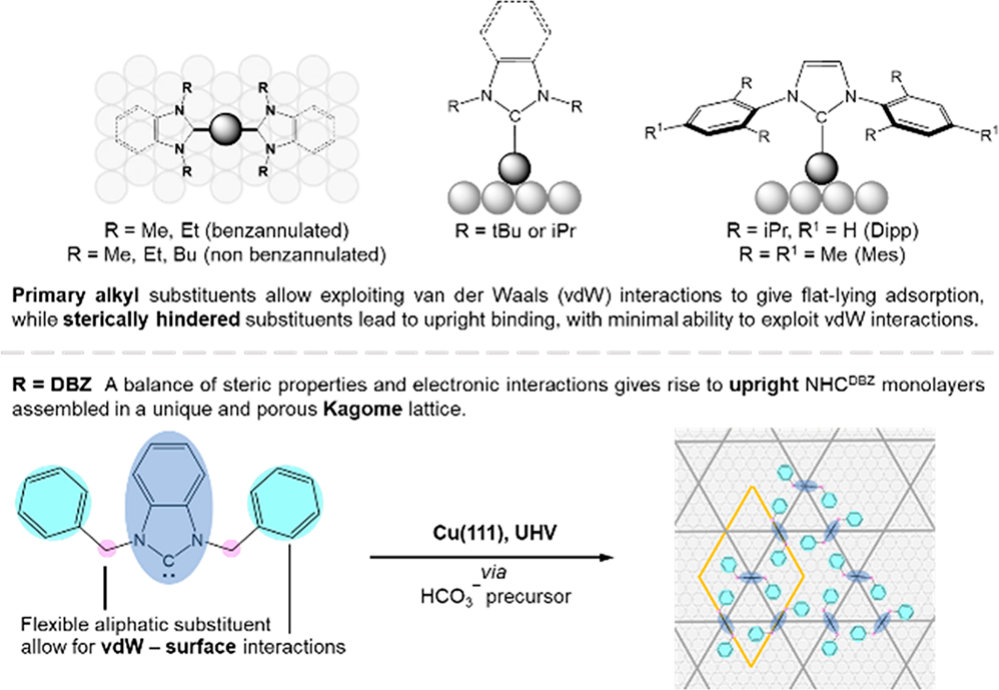
Effects of Exocyclic Nitrogen Substituents (R^i^) on NHC Adsorption Geometry and Assembly Top: Previous work (see text for details). Bottom: This work.

In the case of aryl substituents, van der Waals (vdW) interactions between the aromatic wingtips and the underlying gold surface were proposed to contribute significantly to the adsorption energy.^24^ Recently, it was shown that NHCs with benzylic substituents can serve as initiation points for MOF formation from metal surfaces, but no information was provided on the organization or orientation of the NHC overlayers.^2^

In this work, we provide definitive evidence for the unique binding modes and surface structures obtainable from NHCs bearing flexible benzylic substituents. Cu(111) surfaces are employed because copper and its alloys have numerous applications in catalysis, microelectronics manufacturing and the production of wires, sheets and tubes. Scanning tunneling microscopy (STM), high-resolution electron energy loss spectroscopy (HREELS), and density functional theory (DFT) calculations combine to demonstrate the formation of highly ordered, porous assemblies composed of entirely upright N-heterocycles. The aromatic rings adopt an approximately flat geometry providing additional stabilization via dispersive and weak covalent interactions.^24^ Self-assembly of the benzene rings drives long-range ordering into a Kagome-like lattice,^29−31^ (Scheme 1, bottom).

The hydrogen carbonate salt NHC^DBZ^·H_2_CO_3_ was prepared by benzylation of the parent benzimidazole and ion exchange (see Supporting Information, SI1). NHC overlayers were prepared by heating NHC^DBZ^·H_2_CO_3_ in a solid doser attached to the ultrahigh vacuum systems in direct line of sight to a Cu(111) sample. The resulting overlayers were examined by STM at room temperature (Figure 1).

**Figure 1:**
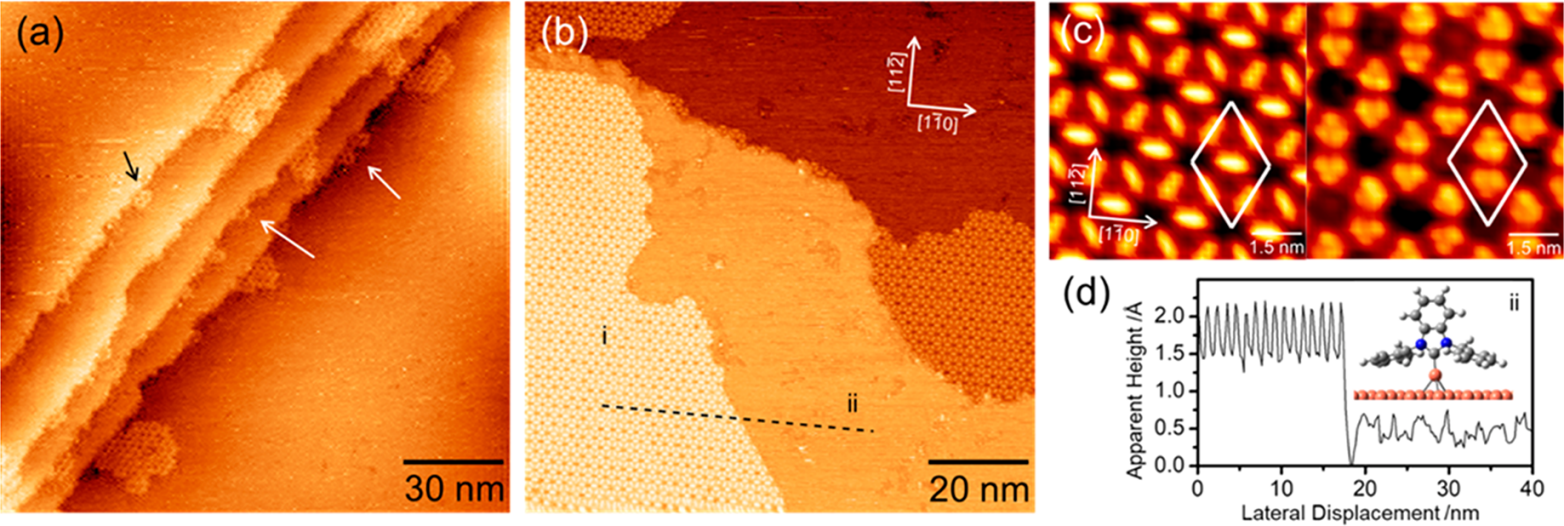
STM images acquired after dosing ∼170 L of NHC^DBZ^/Cu(111) (a) as prepared, room temperature, 150 × 150 nm^2^; (b) annealed to 430 K, 100 × 100 nm^2^; (c) left, magnification of the “i” domain in (b), right, same domain recorded with different scanning parameters, both 7.5 × 7.5 nm^2^; (d) line profile i in (b); all images −1.2 V, 0.25 nA, except (c, right) +1 V, 0.125 nA.

Small domains concentrated in the vicinity of step edges (Figure 1a, arrows) appeared following exposure to 170 Langmuir (L) of NHC^DBZ^ at 300 K. Doses are reported in this way to allow a convenient comparison between different exposures: no corrections have been made for ion gauge sensitivity. Upon annealing to 430 K, much larger ordered islands of the same periodicity are observed (Figure 1b) separated by large areas exhibiting no order. It is likely that the total area of ordered features has increased on annealing. The ordered domains are characterized by a hexagonal arrangement of pores of diameter ∼1 nm. Self-correlation analysis of several STM images reveals a unit cell dimension of 2.1 ± 0.1 nm along ⟨110⟩ directions of the surface (white in Figure 1c), consistent with the formation of a commensurate (8 × 8) overlayer (SI2). The higher contrast features that define the triangular units (Figures 1c, left, −1.2 V bias) are assigned to the benzimidazole backbone. Under different imaging conditions (Figure 1c, right, + 1 V bias), three features whose shape and dimensions are consistent with the benzene rings of the NHC wingtips are observed within each triangular unit. This motif can only be generated if the two benzylic wingtip substituents take up a trans arrangement either side of an upright benzimidazolylidene unit,^32^ which enables them to bridge two different triangular units. The propagation of adsorbates adopting this geometry generates the highly ordered porous domain structure. The cross-section ii in Figure 1b (Figure 1d) shows that, at those specific scanning conditions, the molecular layer has an apparent height of ∼1.5 Å, a value that cannot be taken as a true representation of the geometrical height. This clearly demonstrates that the STM contrast is dominated by electronic factors, as often observed for molecules adsorbed on metallic or semiconducting substrates.^33^ Some of the hexagonal pores appear to house additional electron density. The possibility of copper adatoms being incorporated into the Kagome pores and giving rise to some of the contrast observed in STM cannot be discounted. This is readily expected on Cu(111), rather than on Au(111) where much of the prior work was done.^25^ The ordered domains were found to be susceptible to some disruption whereby a domain present in one image would be perturbed by the STM tip in the next (SI3), especially for tunnelling currents >0.5 nA. This high mobility, coupled with the knowledge that the diffusion barrier for NHC species coordinated to gold adatoms has been computed to be about 1 order of magnitude lower than the diffusion barrier for NHC species bound to atoms in Au(111) terraces,^19^ suggests that the ordered domains consist of NHC^DBZ^ species coordinated to copper adatoms. The incorporation of copper atoms in the self-assembly of other NHCs was reported by Larrea et al.^24^ and has also been demonstrated following the adsorption of carboxylic acids and triazoles on copper surfaces.^34−36^ Likely sources of copper atoms include low coordination sites at step edges and free copper adatoms which are present on low index copper surfaces.^37^ The surface concentrations of adatoms on Cu(111) is known to be low,^36^ so extraction of copper atoms from step-edges is likely to drive the nucleation of molecular islands in the vicinity of steps. This is consistent with data shown in Figure 1a, where initial overlayer formation occurs at step edges. On annealing, islands accommodate further NHCs, and once sufficient thermal energy is available for surface diffusion, large islands propagate into the terrace.

The comparison between the STM image (Figure 2a) and on-surface DFT calculations (Figure 2b) shows a strong agreement, leading to the conclusion that NHC^DBZ^ forms a commensurate (8 × 8) structure on Cu(111), with each NHC^DBZ^ attached to a copper adatom forming Cu-NHC^DBZ^ species which self-assemble to produce ordered structures. In the optimized DFT model, the adsorption sites of the Cu-NHC^DBZ^ species are identified as 3-fold hollow sites, whereas the adsorbate packing arrangement is likely due to the interplay between intermolecular interactions and interactions between the aromatic rings of the benzyl substituents and the Cu surface.

**Figure 2:**
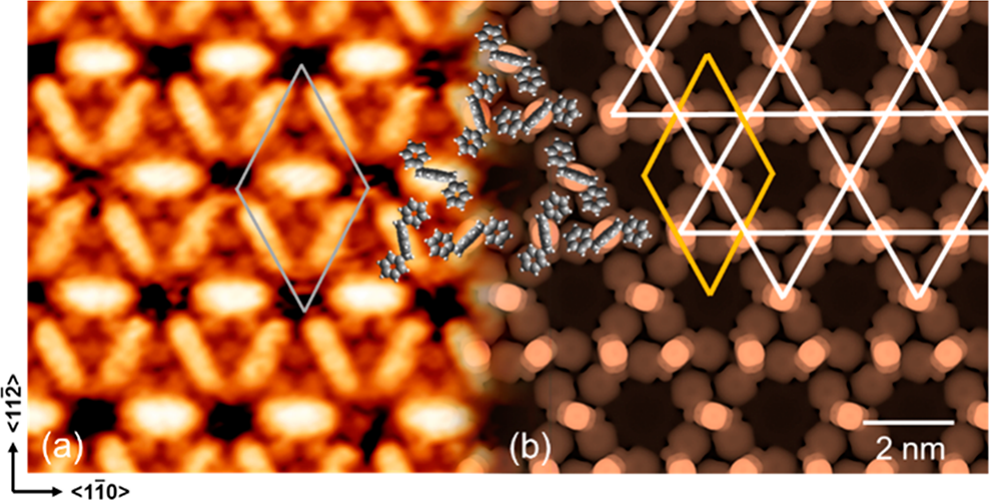
(a) STM topography of NHC^DBZ^/Cu(111), thermal drift corrected, 10 × 10 nm^2^, −1.2 V, 0.25 nA and a (b) simulated STM image at −1.2 V, of the (8 × 8) DFT model; between panels a and b, superposed to scale molecular models; the unit cell is highlighted in both panels (gray/yellow); a Kagome lattice (white) is superposed in panel b.

When modeling a terrace-bound NHC^DBZ^ (i.e., where Cu adatoms are removed), adsorption occurs through the carbene C, which binds to a surface Cu atom (i.e., in the atop position), which is slightly pulled out of the surface; while the arrangement of NHC^DBZ^ species in the plane remains largely unchanged, as the lateral molecule–molecule interactions and phenyl-surface dispersive interactions still remain, their adsorption energy is reduced from 2.9 to 1.1 eV per molecule, clearly indicating a stabilization effect due to the Cu adatoms.

The overall molecular packing closely resembles a Kagome lattice. The relative position of the benzene rings is similar to the molecular separation in the (√7 × √7)R ± 19.1° structure for benzene on Ni(111),^38^ where neighboring adsorbates interleave to minimize repulsion between C–H groups. Inconsistencies between the observed STM images and the DFT model presented in Figure 2 deserve mention. The pores observed in STM images, Figure 2a, are smaller than those predicted by DFT modeling, Figure 2b, which would have arisen from the finite size of the STM tip itself that always makes topographic protrusions appear wider and depressions narrower. The bright feature assigned to the benzimidazole backbone appears larger in the STM image than DFT calculations suggest, which is solely related to a scanning effect. The large pore in the simulated STM image deviates from a perfect hexagonal structure. The most reasonable explanation for the observed images is that the benzimidazole unit and the copper atom to which it is coordinated slide backward and forward on the time scale of the STM image acquisition, giving rise to elongation of the bright features and shrinking of the pores.

No change in the periodicity of the ordered structure is observed following annealing across a wide temperature range, indicating that the Cu-NHC^DBZ^ complexes are thermally stable. TPD studies reported NHC desorption with a *T*_max_ of 550 K.^23^ Desorption and surface mediated decomposition processes compete such that disordered oligomeric species remain on the surface even after annealing to relatively high temperatures (SI4).

The tendency of NHC^DBZ^ to adsorb on Cu(111) with the benzimidazole moiety perpendicular to the surface contrasts to the behavior of *N,N-*dialkylbenzimidazolylidenes (R = Me, Et) and the simpler *N,N*-dialkylimidazolylidenes (R = Me, Et, Bu), all of which form flat-lying M(NHC)_2_ complexes (Scheme 1, top).^6,13,25−28^ We compute that surface-bound *N,N-*dimethylbenzimidazolylidene is 0.45 eV more stable with the benzimidazole moiety parallel rather than perpendicular to the surface (SI5). In the case of NHC^DBZ^, the energy cost associated with the benzimidazole moiety adopting a perpendicular geometry is more than compensated by the interaction of two aromatic N-substituents with the copper surface considering that one molecule of benzene, in an optimized geometry, has an adsorption energy of ∼0.8 eV on Cu(111).^39^ Intermolecular interactions in the overlayer likely provide additional stabilization.

Further insight into the orientation of the NHC overlayers on Cu(111) was provided by HREELS (Figure 3).

**Figure 3:**
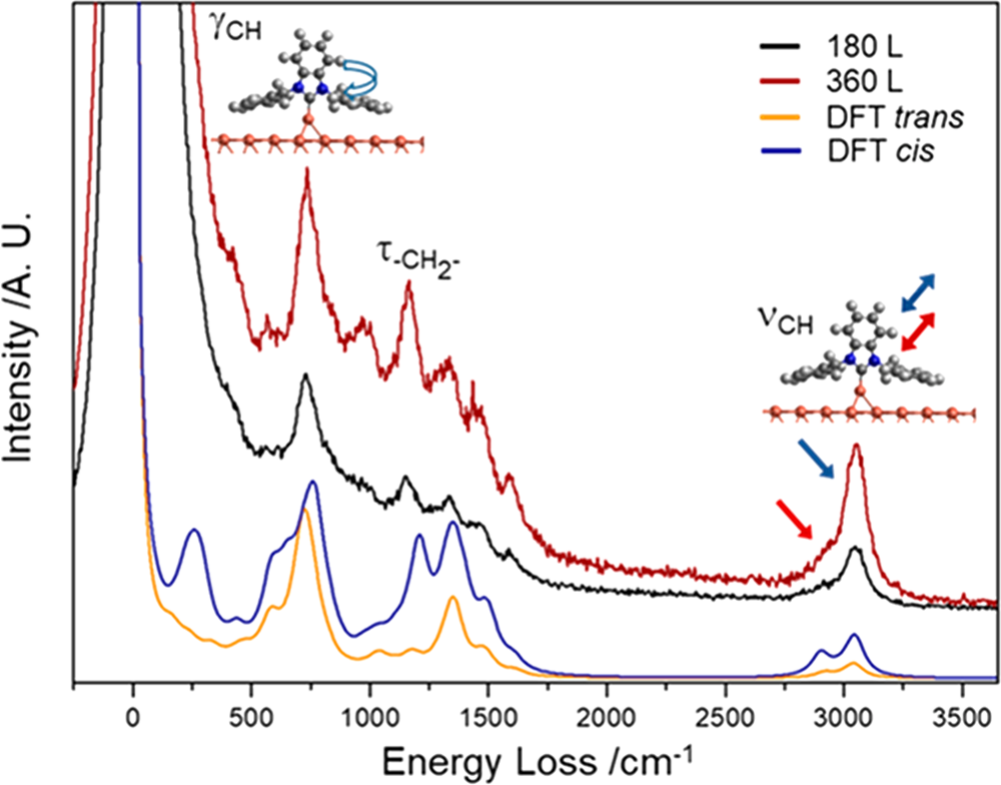
HREEL spectra collected following exposure of the Cu(111) sample to NHC^DBZ^ vapor at ∼2 × 10^–7^ mbar and 300 K at increased coverage: black, 180 L; dark red, 360 L. In orange/blue, the vibrational spectrum of trans/cis conformers calculated via DFT methods. Cartoons illustrate the main vibrations. γ, out of plane bend; τ, twist; ν, stretch.

Spectra were collected as a function of NHC^DBZ^ coverage and compared with the vibrational spectra of both *cis-* and *trans*-NHC^DBZ^ calculated via DFT. The main observed energy losses are attributed to an out-of-plane CH bend mode (γ_CH_) of the phenyl/benzimidazole rings at 730 cm^–1^, a twist of the −CH_2_– at 1145 cm^–1^ (τ_–CH_2_–_) and the aromatic CH stretch (ν_CH_) at 3050 cm^–1^. The latter peak shows a shoulder at ca. 2910 cm^–1^ due to the aliphatic ν_CH_ of the −CH_2_– groups (SI6). A general increase in intensity of all bands is seen with increasing exposure (SI6). An adsorption geometry in which all aromatic rings were parallel to the surface would, by application of the metal surface dipole selection rule, yield a spectrum with an intense γ_CH_ mode at 730 cm^–1^ and a very weak ν_CH_ at 3050 cm^–1^.^25,36,40−42^ Instead, the γ_CH_(730): ν_CH_(3050) ratio indicates that one or more of the aromatic rings of NHC^DBZ^ is not parallel to the copper surface. This is consistent with STM results and unfavorable steric interactions in an “all planar” geometry (SI7). The simulated vibrational spectra of *trans-* and *cis-*NHCs bound to a copper atom are in close agreement with the experimental data and support the geometry proposed in light of the STM measurements. Simulations indicate that the fingerprints of both conformers contribute to the experimental spectra.

DFT was used to compare the adsorption energy of the two conformers of NHC^DBZ^ (SI8). For isolated adsorbates coordinated to copper adatoms, the cis species adsorbed with the heterocycle parallel to the surface was only ∼0.14 eV more stable than the trans species adsorbed with the heterocycle upright. This suggests that *cis*-NHC^DBZ^ may be present on the surface but too mobile to image at 300 K. At higher temperatures, cis species can more readily overcome the activation barrier (calculated to be ∼0.36 eV, SI9) to convert to trans species via rotation of the benzene rings around the −CH_2_– group. This process would then augment the supply of trans species and allow for growth of ordered domains.

On the Cu(111) surface, *trans-*NHC^DBZ^ can assume two enantiomeric configurations (Scheme 2).

**Scheme 2:**
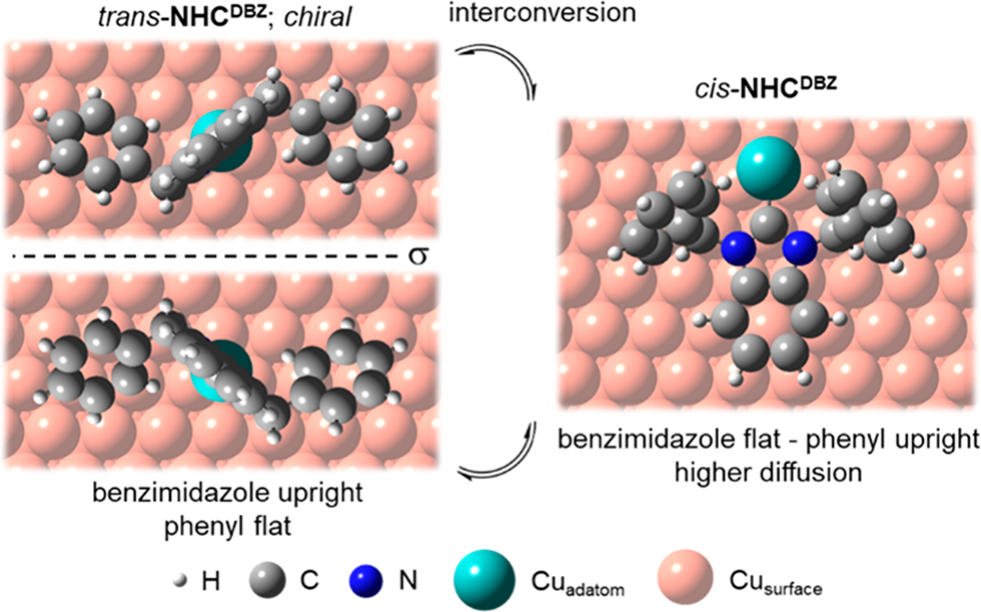
Top Views of NHC^DBZ^ Adsorption Configurations on Cu(111), Highlighting the Chirality of the trans Conformer and the trans/cis Interconversion σ: Mirror plane.

Since the surface is initially achiral, a racemic mixture of *trans*-Cu-NHC^DBZ^ adsorbates exists over the whole surface. We were unable to identify which enantiomeric adsorption complex is present in each domain because the STM images have a higher symmetry than the model predicts, and we were unable to resolve features at domain edges which would help to distinguish enantiomers. If a mixture of enantiomers were present within a domain, one would predict many stacking faults within the molecular arrays or defects consisting of missing features. The lack of such defects suggests that the ordered domains are homochiral (SI10).^43^

In this study, the adsorption behavior of NHC^DBZ^ on Cu(111) was investigated. Cu-NHC^DBZ^ species are formed on adsorption, presumably via extraction of copper atoms from step edges. Highly ordered and extended homochiral domains of Cu-NHC^DBZ^ species are observed in STM images. DFT and STM reveal that the adsorbate forms ordered (8 × 8) arrays with three Cu-NHC^DBZ^ species per unit cell. HREELS, DFT, and STM show that the benzimidazole moiety adsorbs perpendicular to the surface with the phenyl rings of the benzylic substituents lying flat and controlling the ordering of the overlayer into a Kagome-like lattice. These ordered overlayers generate well-defined nanosized pores, which are potentially of considerable utility in applications where confinement effects on this length scale are important. Moreover, when functionalized at the benzene end, the upright geometry of the benzimidazole opens the possibility to grow 3D structures.

## Supporting Information Available

Synthesis of NHC^DBZ^, additional STM images, additional DFT calculations, additional vibrational spectroscopy, experimental and computational details, and additional references (PDF)

## Notes

The research data underpinning this publication can be accessed at https://doi.org/10.17630/3ceb4033-456e-49bf-b29c-c99314a1fa07.
