# Supplementary material for: Highly Ordered N-Heterocyclic Carbene Monolayers on Cu(111)
Source: J Phys Chem Lett. 2022 Feb 24;13(8):2051–6. doi: 10.1021/acs.jpclett.1c04073 (PMC9007529; doi:10.1021/acs.jpclett.1c04073)
Supplement: Supplementary file 1 — jz1c04073_si_001.pdf [file jz1c04073_si_001.pdf]

Supporting Information for

Highly Ordered *N*-Heterocyclic Carbene Monolayers on Cu(111)

Eloise Angove[1], Federico Grillo[1], Herbert A Früchtl[1], Alex J. Veinot[2], Ishwar Singh[2], J. Hugh Horton[2], Cathleen M Crudden[2,3], Christopher J Baddeley[1*]

1. EaStCHEM School of Chemistry, University of St Andrews, North Haugh, St Andrews, Fife, KY16 9ST
2. Department of Chemistry, Queen's University, 90 Bader Lane, Kingston, Ontario, Canada, K7L 3N6
3. Institute of Transformative Bio-Molecules, ITbM-WPI, Nagoya University, Nagoya, Chikusa 464-8601, Japan

* email cjb14@st-andrews.ac.uk

Contents

SI1. Synthesis of NHC^{DBZ}

SI2. Self-correlation analysis of STM images

SI3. Tip-induced layer modification

SI4. STM domains vs coverage and vs annealing

SI5. DFT calculations of a single NHC^{dMe} molecule adsorbed on Cu(111) through a Cu adatom

SI6. HREELS assignments and spectra evolution with increasing coverage and annealing

SI7. Cu-NHC^{DBZ} hypothetical "all flat" geometries

SI8. DFT calculations of a single NHC^{DBZ} molecule adsorbed on Cu(111) through a Cu adatom

SI9. On-surface energy barriers isomerisation NEB calculations

SI10. *cis/trans* models and homochiral surface domains

S11. Experimental and computational details

S12. References

SI1. Synthesis of NHC^{DBZ}

Benzimidazole (98 %), benzyl chloride (99 %), potassium carbonate (99 %), acetonitrile (99.9 %, HPLC grade), and diethyl ether (99.0 %, anhydrous) were obtained from Sigma-Aldrich and used as received. Amberlyst A26 hydroxide resin was obtained from Sigma-Aldrich and converted to HCO_3^- anion exchange resin following a previously reported literature procedure [1].

The benzimidazolium hydrogen carbonate salt was prepared from its corresponding chloride salt, using a freshly prepared anion exchange resin, following previously reported literature procedures [2], spectroscopic data were consistent with the literature. The completeness of anion exchange for hydrogen carbonate salts were verified using elemental analysis prior to surface deposition.

SI2. Self-correlation analysis of STM images.

Figure S1 shows an example of the estimation of the unit cell size trough self-correlation and radial distribution analysis of the STM images, using the WSxM software package [3]. Using an image with a large island present, Figure S1(a), an area of 25 × 25 nm^2 was selected consistently, Figure S1(b). This area was flattened, global planed and then a self-correlation filter was applied, Figure S1(c). Finally, a radial average distribution filter was applied resulting in Figure S1(d), where the distance to the first peak represents the average spacing of the unit cell. Multiple images of different sizes were selected for this process, as shown in Table S1.

Table S1: self-correlation and radial distribution analysis

Original frame size /(nm × nm)	Radius /nm
42 × 42	2.105
50 × 50	2.084
50 × 50	2.085
80 × 80	2.127
80 × 80	2.132
100 × 100	2.152
100 × 100	2.152
100 × 100	2.178
130 × 130	2.125
150 × 150	2.066
150 × 150	2.091
average	2.118

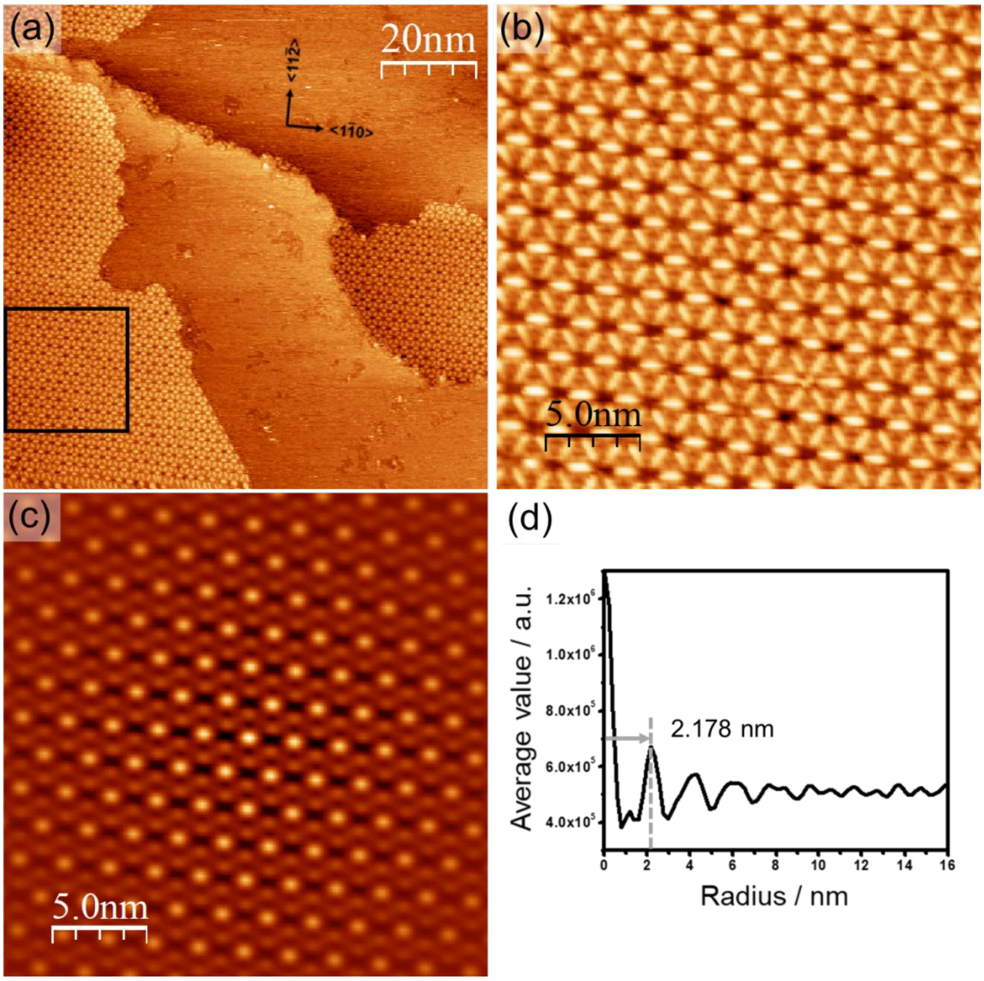

Figure S1. Example of self-correlation and radial distribution analysis: (a) original frame, 100 × 100 nm^2; (b) 25 × 25 nm^2 area selection; (c) self-correlation filter applied to (b); (d) radial distribution filter applied to (c).

SI3.Tip-induced domains modification.

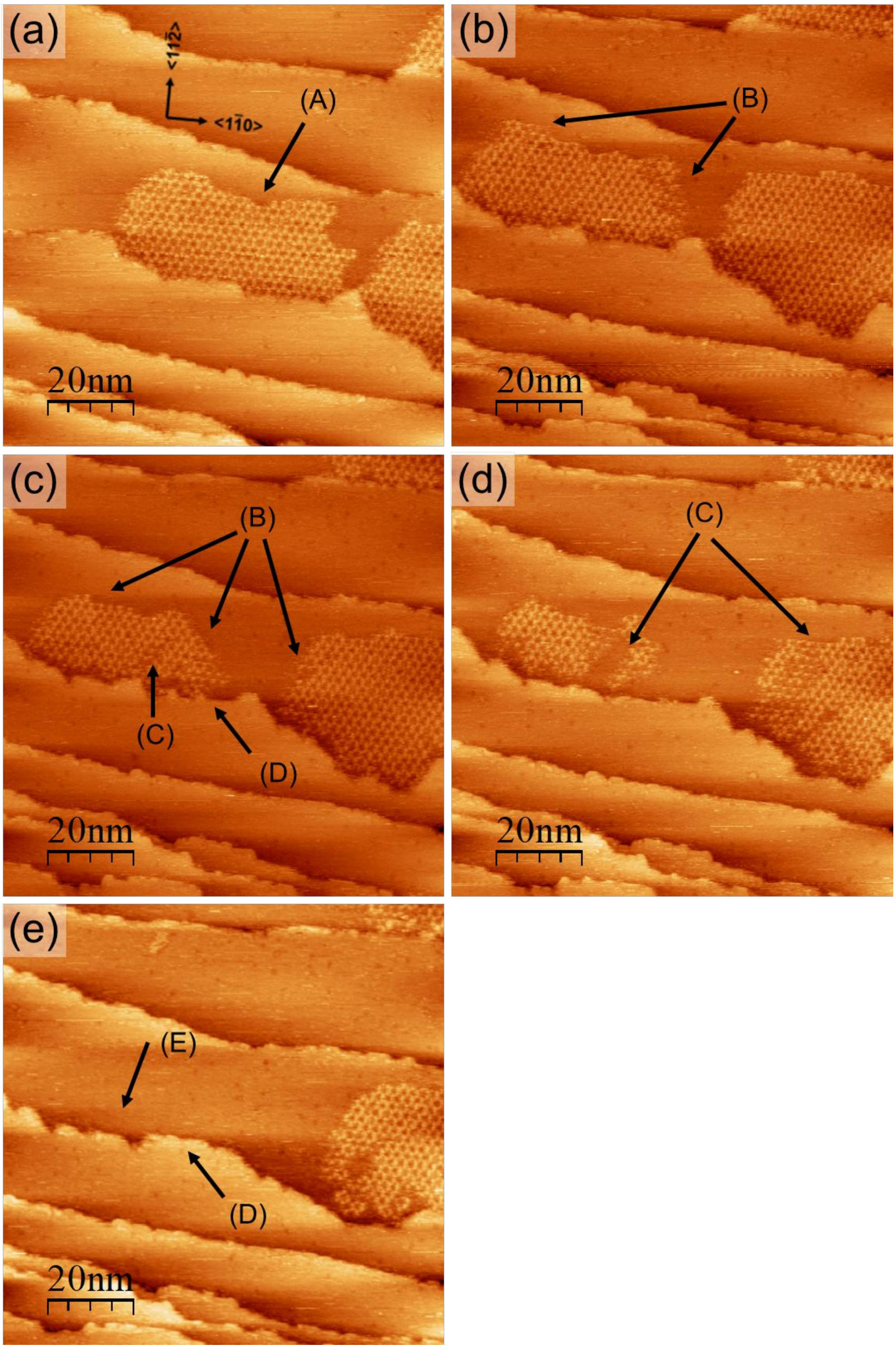

Figure S2. Consecutive STM topographs showing tip induced NHC^{DBZ} islands' modification on Cu(111),(a – e) ~170 L, 400 K, 100 × 100 nm^2, -1.2 V, 0.5 nA. (A) indicates adsorbate's island, (B) defects at island's edge, (C) defects within island, (D) modification at the step edge and (E) disappearance of island.

SI4.STM domains vs coverage and vs annealing

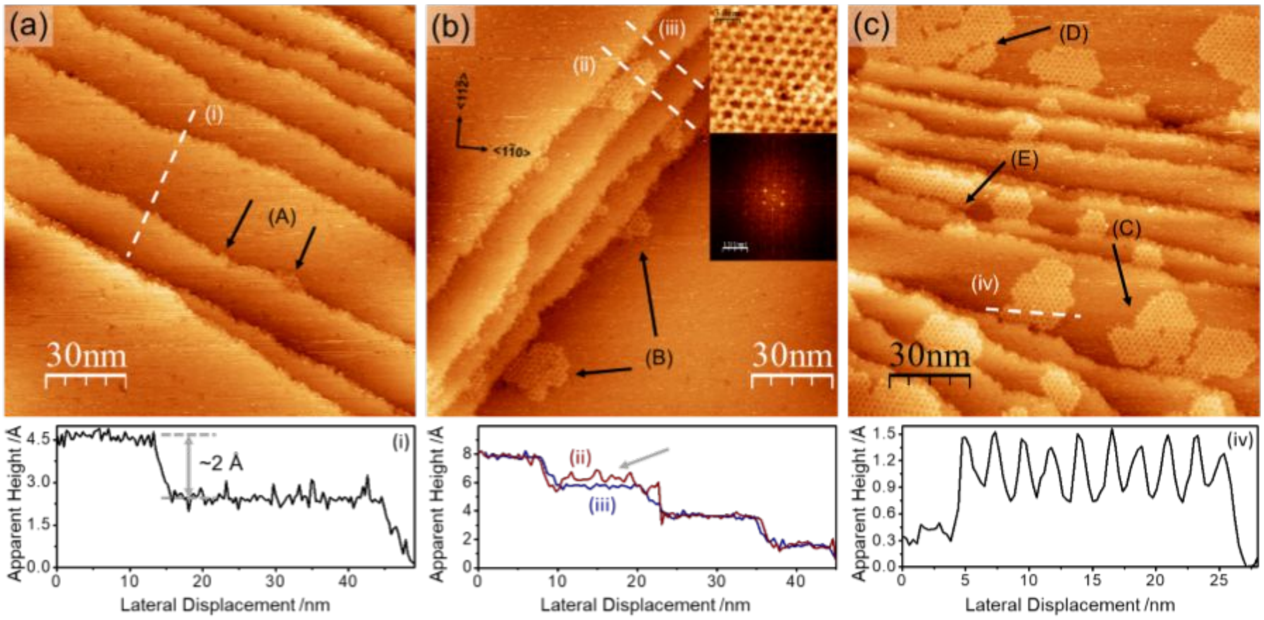

Figure S3. STM images showing the growth of NHC^{DBZ}islands on Cu(111) following exposure to NHC^{DBZ}vapour at 1.5 – 2 × 10^{-8} mbar and 300 K; (a) ~50 L, 150 × 150 nm^2, - 1.2 V, 0.25 nA; (b) same as manuscript figure 1a~85 L, 150 × 150 nm^2, - 1.2 V, 0.25 nA; in the inset magnification of an adsorbate island 19 × 19 nm^2, -1.0 V, 0.15 nA, and corresponding fast Fourier transform filtering; (c) ~170 L, 150 × 150 nm^2, - 1.1 V, 0.10 nA; (i - iv) line profiles as in (a - c); arrows (A - E) indicate adsorbate's islands.

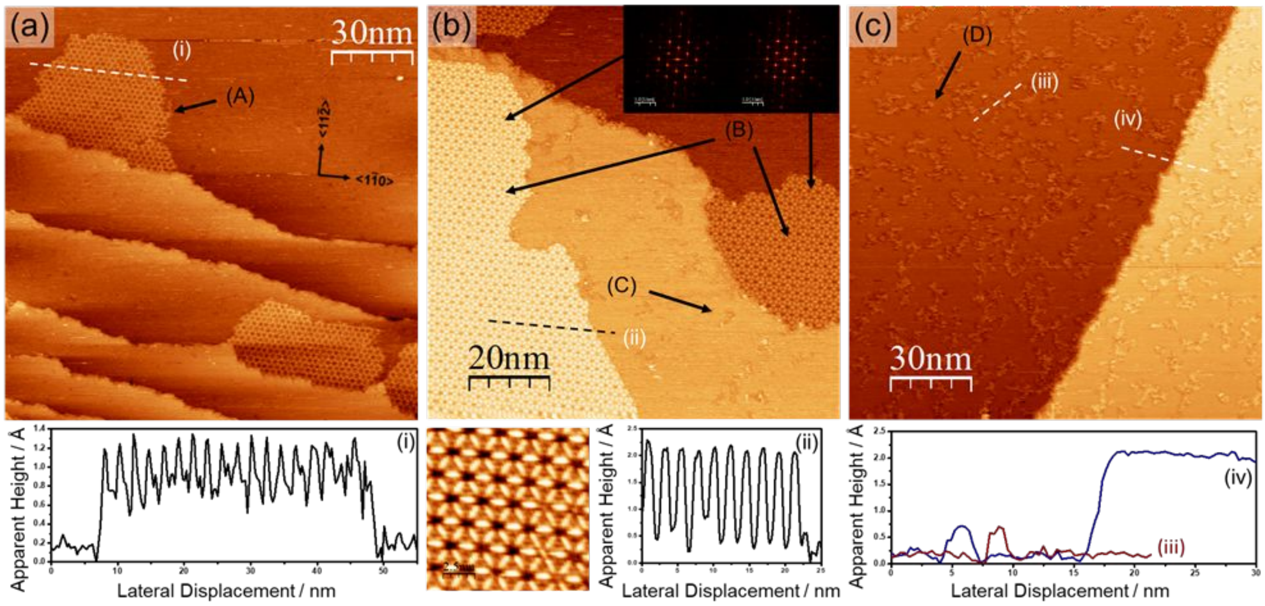

Figure S4. STM images acquired after annealing of a preparation of ~170 L of NHC^{DBZ}/Cu(111) at (a) 400 K, 150 × 150 nm^2; (b) same as manuscript figure 1b, 430 K, 100 × 100 nm^2 and Fast Fourier Transform filtering for both islands, 12 × 12 nm^2; (c) 550 K, 150 × 150 nm^2; (i - iv) line profiles as in (a - c). All images -1.2 V, 0.25 nA; (A) indicates defects at island's edge, (B) well-ordered and defect-free islands, (C-D) defects on terraces of different origin.

SI5. DFT calculations of a single NHC^{dMe} molecule adsorbed on Cu(111) through a Cu adatom

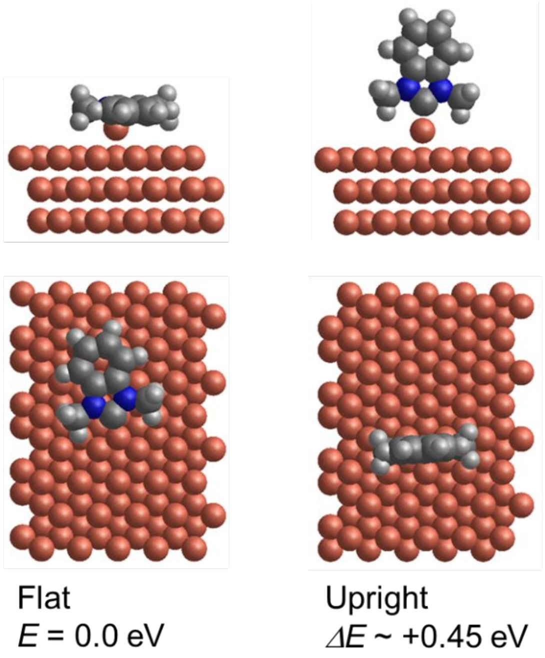

Figure S5. DFT calculations of a single NHC^{dMe} molecule adsorbed on Cu(111) through a Cu adatom. Colour code: H, light grey; C, dark grey; N, blue; Cu, orange.

SI6. HREELS assignments and spectra evolution with increasing coverage and annealing

Table S2: HREELS energy loss assignments compared with calculated frequencies as in manuscript Figure 3; values in cm^{-1}.

Mode	Measured Energy Loss	Calculated *cis*	Calculated *trans*
ν CH aromatic	3050	3045	3040
ν CH aliphatic	2910	2910	2925
ν CC breathing benzimidazole	1590	1595	1615
δ CH benzimidazole	1445	1485	1475
δ CH benzimidazole+ ν CN (N-CH_2-)	1330	1355	1350
τ –CH_2– + δ CH	1145	1210	1175
breathing + δ CH benzimidazole	950	1050	1035
γ CH (phenyl / benzimidazole)	730 / 815 s	605 s/ 665 s/ 760	590 s/ 725
τ benzimidazole	570	440	475
γ benzyl + δ benzimidazole	415	265	325

ν stretch, δ in-plane bend, γ out-of-plane bend, τ twist, s shoulder

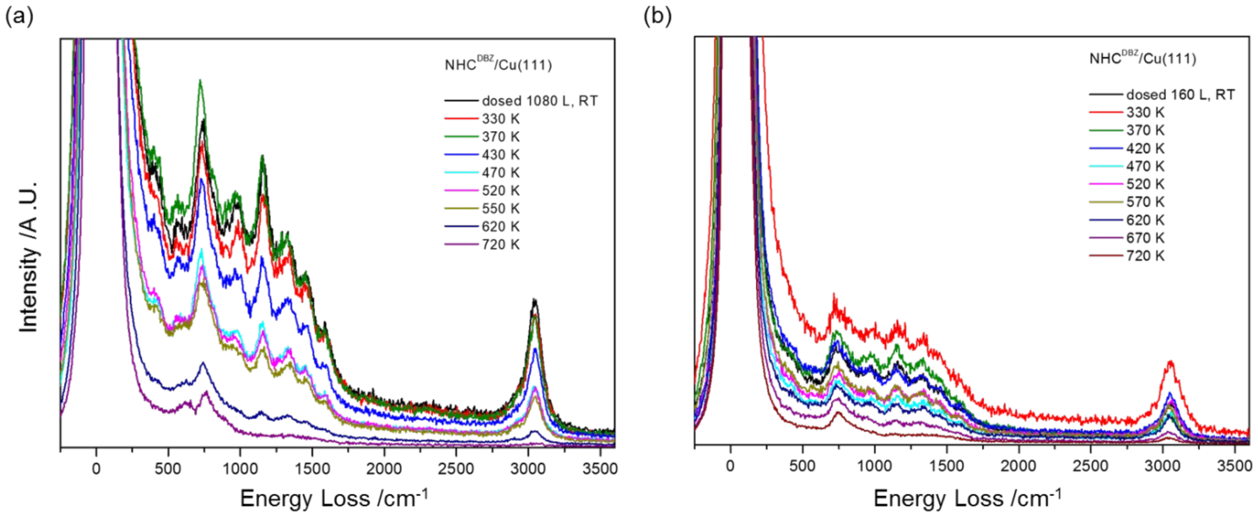

Figure S6. HREEL spectra collected upon annealing a cumulative exposure of (a) 1080 L NHC^{DBZ}/Cu(111) preparation to the temperatures indicated in figure and (b) 160 L NHC^{DBZ}/Cu(111). Spectra are on a common intensity scale. The better signal to noise ratio of the high coverage preparation (a) indicates a higher degree of crystallinity of the adsorbed molecular layer.

SI7. Cu-NHC^{DBZ} hypothetical "all flat" geometries

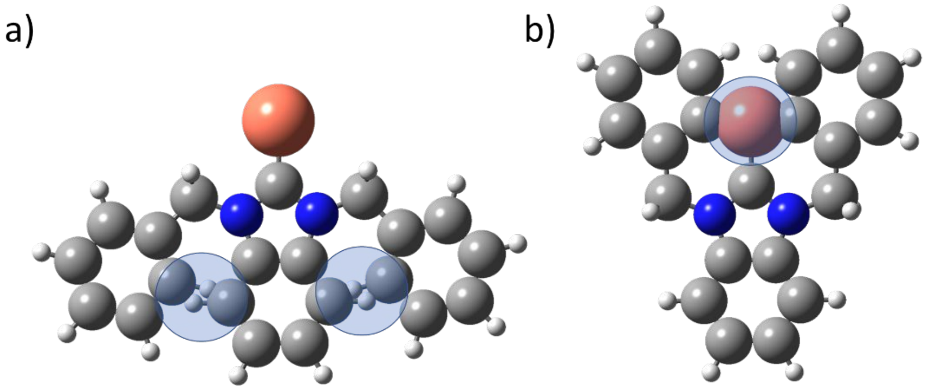

Figure S7. Cu-NHC^{DBZ} hypothetical "all flat" geometries, whereby the *N*-heterocycle and the phenyl rings lay on the same pane, showing several steric hindrances (blue shades); a) C-H/C-H hindrance; b) C-H/Cu hindrance. Colour code: H, light grey; C, dark grey; N, blue; Cu, orange.

SI8. DFT calculations of a single NHC^{DBZ} molecule adsorbed on Cu(111) through a Cu adatom

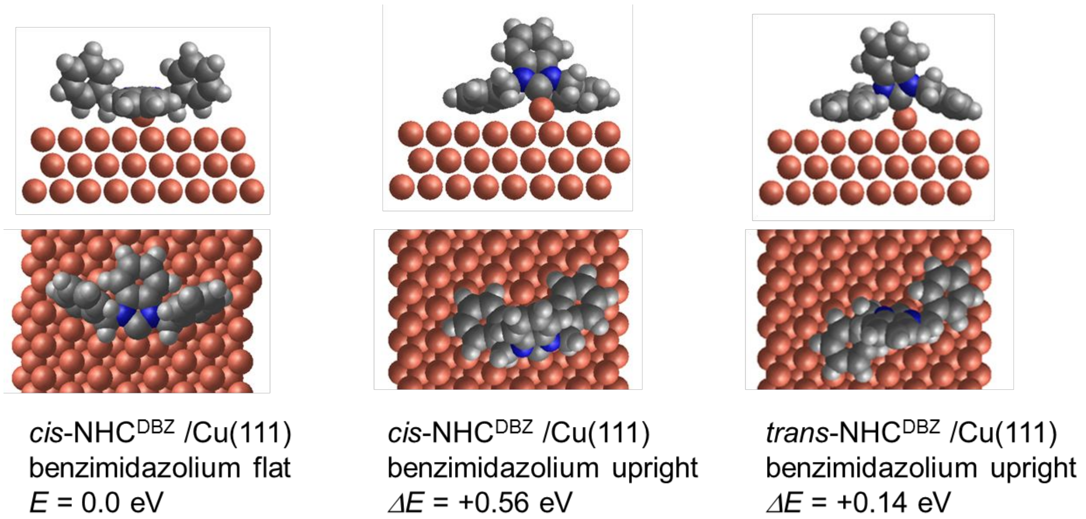

Figure S8. DFT calculations of a single NHC^{DBZ} molecule adsorbed on Cu(111) through a Cu adatom. Colour code: H, light grey; C, dark grey; N, blue; Cu, orange.

SI9. On-surface energy barriers isomerisation NEB calculations

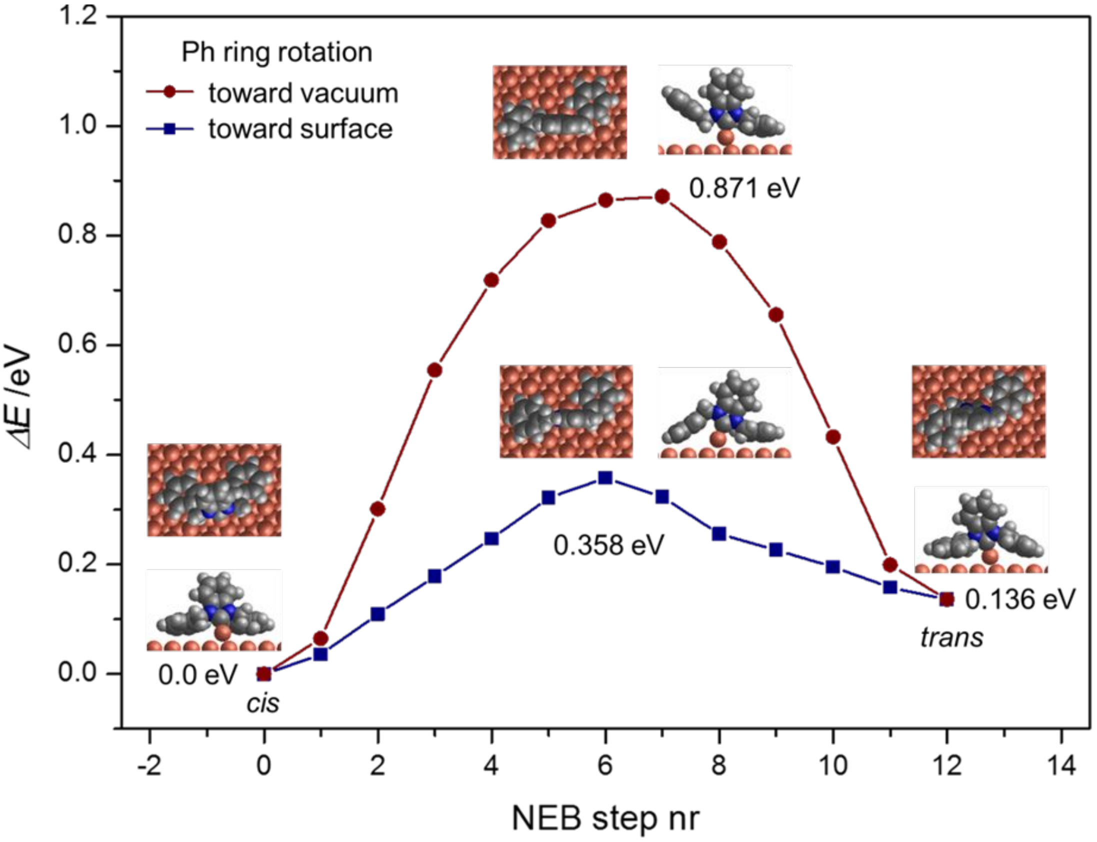

Figure S9. On-surface energy barriers isomerisation NEB calculations. Colour code: H, light grey; C, dark grey; N, blue; Cu, orange.

SI10. *cis/trans* models and homochiral surface domains

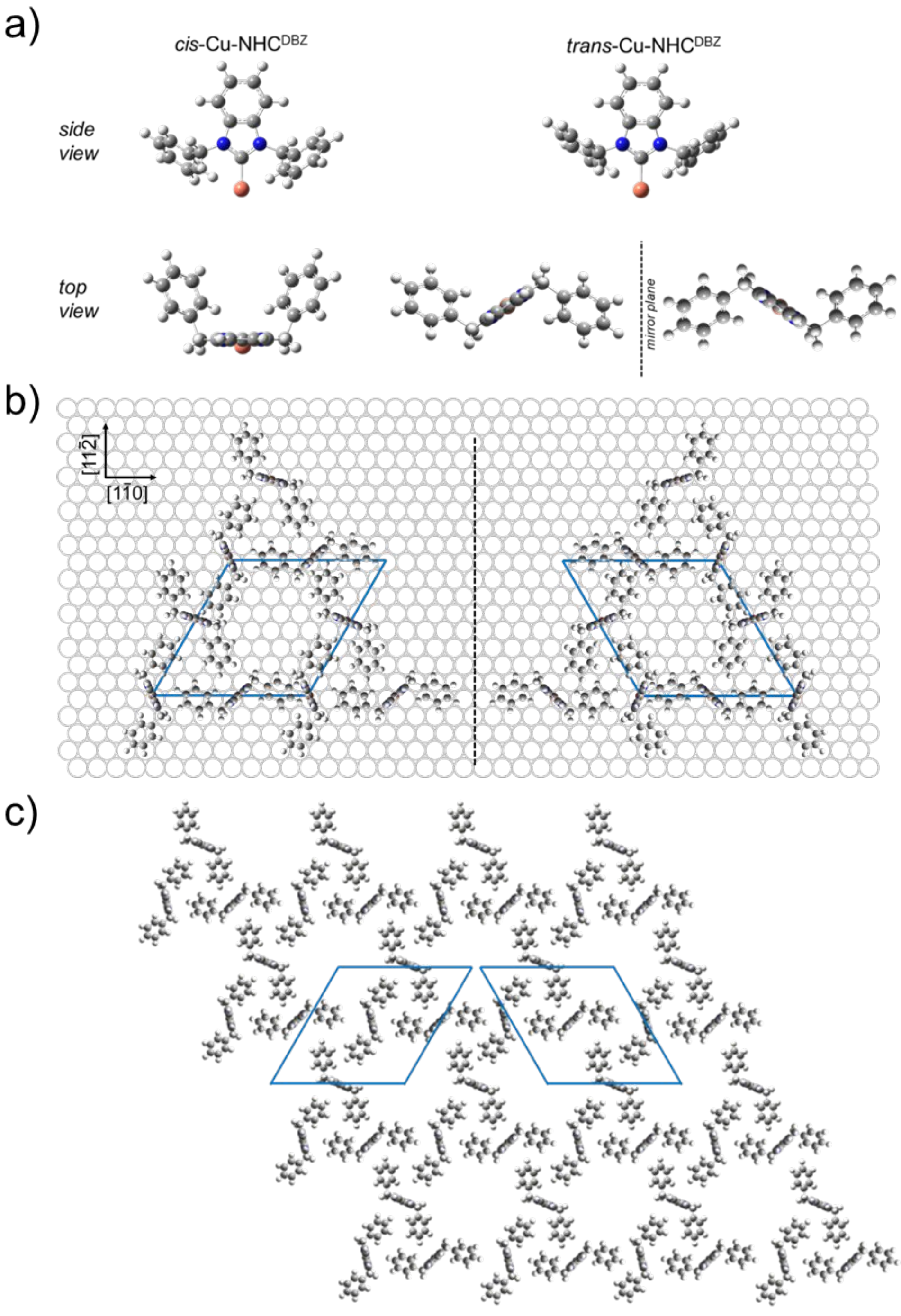

Figure S10. a) Top-view and side-view of *cis*- and *trans*-Cu-NHC^{DBZ}; the chiral nature of *trans*-Cu-NHC^{DBZ} is highlighted; b) upon adsorption on Cu(111), chiral domains of *trans*-Cu-NHC^{DBZ} can form; c) a graphical demonstration that without evaluating the domain's edge, the absolute chirality cannot be determined. Colour code: H, light grey; C, dark grey; N, blue; Cu adatom, orange; Cu surface, open circles.

S11. Experimental and computational details

STM experiments were conducted in a UHV chamber (variable temperature-STM, Omicron) with a base pressure of 1 × 10^{-10} mbar. The chamber was additionally equipped with low energy electron diffraction (SpectaLEED optics, Omicron). The Cu(111) single crystal was subjected to cleaning cycles of Ar^+ bombardment and annealing to ~800 K until a sharp (1 × 1) LEED pattern was observed, and large atomically flat terraces were imaged by STM. The STM images were recorded in constant current mode, at room temperature (~300 K), using an electrochemically etched tungsten tip. STM images were analyzed using the WSxM software [3]. The temperature was measured through a K-type thermocouple attached to the sample holder, in close proximity of the sample; temperatures are estimated to be within +/- 5 K.

HREELS (VSW HIB 1001 double-pass spectrometer) experiments were carried out in a separate UHV system consisting of a preparation chamber equipped with surface preparation facilities and LEED, with a base pressure lower than 1 × 10^{-10} mbar, and a separate chamber housing the HREEL spectrometer, with a base pressure lower than 5 × 10^{-11} mbar. Measurements were collected operating the spectrometer in the specular geometry ($\theta_i = \theta_f = 45°$), with a primary beam energy of 5 eV and a typical elastic peak resolution of ~50 cm^{-1} (6.2 meV full width at half maximum, FWHM). The temperature was measured through a K-type thermocouple in direct contact with the sample; temperatures are estimated within +/- 5 K. From chemical changes observed investigating standard systems, a discrepancy of 15-20 K between STM and HREELS chambers is typically observed; this was taken into account during data analysis and interpretation. The Cu(111) single crystal was cleaned through cycles of Ar^+ bombardment and annealing to ~800 K until a sharp (1×1) LEED pattern and a featureless HREEL spectrum were observed. Typically, the background pressure increased to ~2 × 10^{-7} mbar during deposition of NHC^{DBZ}.

NHC^{DBZ} dosing was performed via chemical vapor deposition (CVD), from its hydrogen carbonate salt, NHC^{DBZ}•H_2CO_3, by electrical heating the compound contained in a borosilicate capillary, mounted in a differentially pumped doser isolated from the main chamber by a gate valve. During dosing, the Cu(111) sample, kept at 300 K, is rotated to ensure line of sight to the NHC^{DBZ} crucible. Typically, the background pressure increased to ~1.5 × 10^{-8} mbar during deposition of NHC^{DBZ}. No evidence for the presence of carbonate species was found when dosing, in line with the assumption that, on warming the solid sample, the NHC^{DBZ} is released into the gas phase and any H_2O and CO_2 produced in this process does not stick to the Cu(111) surface at 300 K.

Periodic calculations were carried out using the VASP software [4]. The PBE functional [5] with Grimme's D3 van der Waals correction [6] with Becke-Johnson damping [7], PAW

pseudopotentials [8] and a plane-wave cut-off of 500 eV was employed. The surface was modelled as a slab of three copper layers, of which the lower two were kept at the optimised bulk geometry. Calculations on either hexagonal (8×8) unit cell contained three molecules. Calculations involving a single molecule used a rectangular unit (5×8) cell. A vacuum gap of 15 Å was added between the slabs to accommodate the adsorbate and prevent unphysical interactions in the direction orthogonal to the surface. Simulated STM images were created using the Tersoff-Hamann approximation [9], as implemented in the p4vasp package.

DFT gas phase geometrical optimisation and calculations of vibrational frequencies were carried out using the Gaussian09 software package [10], using the B3LYP functional and 6-311+G(d) basis set, as implemented in the software. Vibrational spectra energy scales were corrected according to the formula proposed by Kasahara *et al.* [11] to compensate for the overestimation due to the functional/basis set combination. For an easier comparison with HREELS data, calculated spectra were convoluted with Gaussian functions having FWHM of 50 cm^{-1}; to simulate the decrease in sensitivity at increased energy loss typical of HREELS, empirically, each intensity was divided by its respective frequency.

S12. References

(1) Crudden, C. M.; Horton, J. H.; Narouz, M. R.; Li, Z.; Smith, C. A.; Munro, K.; Baddeley, C. J.; Larrea, C. R.; Drevniok, B.; Thanabalasingam, B. Simple direct formation of self-assembled N-heterocyclic carbene monolayers on gold and their application in biosensing. *Nat. Commun.* **2016**, *7*, 12654.

(2) Narouz, M. R.; Osten, K. M.; Unsworth, P. J.; Man, R. W. Y.; Salorinne, K.; Takano, S.; Tomihara, R.; Kaappa, S.; Malola, S.; Dinh, C.-T.; Padmos, J. D.; Ayoo, K.; Garrett, P. J.; Nambo, M.; Horton, J. H.; Sargent, E. H.; Häkkinen, H.; Tsukuda, T.; Crudden, C. M. N-heterocyclic carbene-functionalized magic-number gold nanoclusters. *Nat. Chem.* **2019**, *11*, 419-425.

(3) Horcas, I.; Fernández, R.; Gómez-Rodríguez, J. M.; Colchero, J.; Gómez-Herrero J.; Baro, A. M. WSxM: A software for scanning probe microscopy and a tool for nanotechnology, *Rev. Sci. Instrum.* **2007**, *78*, 013705.

(4) Kresse, G.; Furthmüller, J. Efficient iterative schemes for ab initio total-energy calculations using a plane-wave basis set, *Phys. Rev. B - Condens. Matter Mater. Phys.* **1996**, *54*, 11169–11186.

(5) Perdew, J. P.; Burke, K.; Ernzerhof, M. Generalized gradient approximation made simple, *Phys. Rev. Lett.* **1996**, *77*, 3865–3868.

(6) Grimme, S.; Antony, J.; Ehrlich, S.; Krieg, H. A consistent and accurate ab initio parametrization of density functional dispersion correction (DFT-D) for the 94 elements H-Pu, *J. Chem. Phys.* **2010**, *132*, 154104.

(7) Grimme, S.; Ehrlich, S.; Goerigk, L. Effect of the damping function in dispersion corrected density functional theory, *J. Comput. Chem.* **2011**, *32*, 1456-1465.

(8) Joubert, D. From ultrasoft pseudopotentials to the projector augmented-wave method, *Phys. Rev. B - Condens. Matter Mater. Phys.* **1999**, *59*, 1758–1775.

(9) Tersoff, J.; Hamann, D. R. Theory of the scanning tunneling microscope. *Phys. Rev. B* **1985**, *31*, 805–813.

(10) Frisch, M. J.; Trucks, G. W.; Schlegel, H. B.; Scuseria, G. E.; Robb, M. A.; Cheeseman, J. R.; Scalmani, G.; Barone, G.; Mennucci, B.; Petersson, G. A. *et al.*, Gaussian 09, Revision D.01, Gaussian, Inc., Wallingford CT, 2013.

(11) Kasahara, T.; Shinohara, H.; Oshima, Y.; Kadokura, K.; Uriu, Y.; Ohe, C.; Itoh, K. Infrared reflection absorption spectroscopic studies on the adsorption structures of dimethyl sulfide and methyl ethyl sulfide on Ag(110) and Cu(110). *Surf. Sci.* **2004**, *558*, 65–79.
